# Experimental Investigation of Reaction-Induced Pressure Perturbations in PTFE/Al Composites During Shock Compression

**DOI:** 10.3390/ma18184267

**Published:** 2025-09-12

**Authors:** Weixi Tian, Wei Du, Zhenwei Zhang, Jian Pan, Chunxu Pang, Chuanting Wang, Lei Guo, Yuan He, Yong He

**Affiliations:** 1 School of Mechanical Engineering, Nanjing University of Science and Technology, Nanjing 210094, China; tianweixi@njust.edu.cn (W.T.); wdu@njust.edu.cn (W.D.); zwzhang1996@njust.edu.cn (Z.Z.); ctwang@njust.edu.cn (C.W.); guolei@njust.edu.cn (L.G.); 2 Xi’an Modern Control Technology Research Institute, Xi’an 710065, China; panjian0903@163.com (J.P.); hnet2003@21cn.com (C.P.)

**Keywords:** reactive materials, shock compression, initiation, shock-induced reaction

## Abstract

In this study, reaction mechanisms of polytetrafluoroethylene/Al materials under shock compression were investigated. The reaction-induced pressure perturbations in PTFE/Al materials were identified by comparing pressure profiles with those of inert PTFE/LiF counterparts. The pressure rebounded to a range of 10.2–16.9 GPa under an incident shock pressure range of 11.5–22.6 GPa. The pressure perturbation amplitude induced by reaction gradually attenuated with increasing propagation distance. The delay time between the observed pressure perturbations and the incident shock front arrival ranged from 0.84 to 1.71 μs and showed a decreasing trend with increasing incident shock pressure and decreasing aluminum particle size. The results suggest that the reaction ignition and energy release of PTFE/Al materials change from closely following the shock front to being delayed by hundreds of microseconds behind the shock front when shock compression intensity decreases from GPa to MPa levels.

## 1. Introduction

Polytetrafluoroethylene/aluminum (PTFE/Al) composite, as typical reactive materials (RMs), have broad application prospects in military, aerospace, and energy fields due to their ideal energy density, excellent inert characteristics, and superior mechanical strength. These materials remain chemically inert under normal conditions but can be triggered to react and release substantial chemical energy under intense shock loading. Researchers have conducted extensive studies on their preparation processes, mechanical properties, shock reaction characteristics, and engineering applications [1,2,3,4].

The mechanisms of initiation and reaction under shock compression are critical issues in RMs, with temperature rise being considered the dominant factor controlling initiation and reaction processes. Theoretically, researchers have analyzed various heating sources, including shock adiabatic heating under strong shock conditions [4,5], frictional heating between fracture surfaces under low-velocity impact conditions [6], and localized temperature concentration caused by crack tips and shear bands during crack propagation in quasi-static compression processes [7,8]. Some numerical simulations have conducted more in-depth studies on the thermodynamic response of materials at the mesoscale [9,10,11]. Experimentally, Differential Scanning Calorimetry and Thermogravimetric Analysis have been employed to analyze thermochemical reaction mechanisms [12,13]. Various experiments have been conducted to investigate the reaction behavior and influencing factors of materials, including rod impact tests [14,15], split Hopkinson pressure bar (SHPB) tests [6,16], quasi-confined container tests [4,17], drop weight tests [18,19], and explosive loading [16,20]. Experimental results indicate that aluminum particle size, materials formulation ratio, and loading intensity affect reaction ignition and energy release intensity.

Since the shock reaction of PTFE/Al occurs under extreme loading conditions, limitations in testing techniques make it difficult to directly obtain characteristics within the shock compression reaction zone. In some low- or medium-velocity impact tests, such as Taylor impact tests [21] and SHPB tests [22], flame flash observation is typically used to determine the initiation and intensity of reactions in RMs. However, as an indirect macroscopic reaction criterion, flame flash is easily affected by observation conditions and subjective factors, to obtain approximate reaction initiation times. Embedded manganin foil gauge arrays enable monitoring of the internal pressure–time profiles in materials under shock compression [23]. In studies of shock initiation in explosives [24,25], pressure–time histories at multiple locations within the explosive are obtained using manganin gauge arrays, allowing for analysis of reaction behavior and initiation mechanism. Guo and Mao used gas guns and explosive lenses as loading devices and analyzed reaction behavior of PTFE/Al composites through different attenuation characteristics of measured pressure curves [16,20]. Due to the lack of inert counterparts as control groups, the identification of reaction-induced pressure perturbations in shock pressure curves in these experiments was empirical.

Lithium fluoride (LiF), due to its mechanical property characteristics similar to aluminum powder, has been widely adopted as an inert substitute reference for aluminum powder in studies of aluminum-containing explosive detonation performance [26,27]. It has been reported that PTFE/LiF exhibits mechanical properties similar to PTFE/Al and remains stable and inert during shock compression, making it suitable as an inert counterpart [28].

In this study, explosive shock experiments referring to [16,20] were improved by introducing PTFE/LiF as control groups for Al-containing reactive materials. Pressure–time profiles inside materials under shock compression were obtained, and reaction-induced pressure perturbations were identified by comparing pressure profiles with those of inert counterparts. The effects of different shock pressures and aluminum powder particle sizes on reaction initiation behaviors were investigated. This study provides experimental evidence for understanding the reaction initiation mechanism of reactive materials under shock compression.

## 2. Methods

### 2.1. Preparation of Specimen

**Figure 1:**
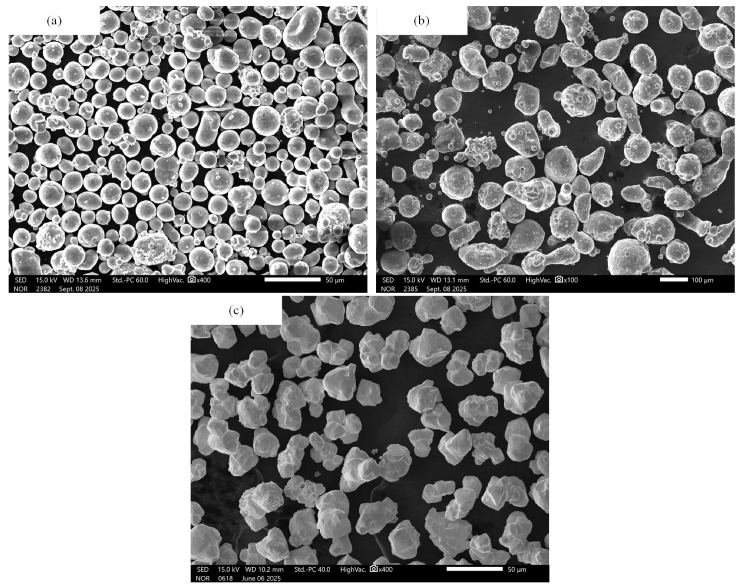
Micro morphology of raw filler particles: (**a**) coarse spherical Al particles; (**b**) fine spherical Al particles; (**c**) polyhedral LiF particles.

As shown in Table 1, this study prepared three types of fluoropolymer-matrix composite samples, including PTFE/Al (PA-1, PA-2) and PTFE/LiF (PF). Each component was mixed according to predetermined mass ratios. The constituent powders were mixed according to specific mass ratios. Figure 1 shows representative scanning electron microscopy (SEM) images of raw filler particles used in the study. The SEM images were acquired using a microscope (JSM-IT00HR, JEOL Ltd., Tokyo, Japan). The two sizes of aluminum particles are approximately spherical with relatively uniform particle size distributions, having average particle sizes of 25 and 75 μm, respectively. The LiF particles are irregular and polyhedral in shape, and the particle size is about 25 μm.

**Figure 2:**
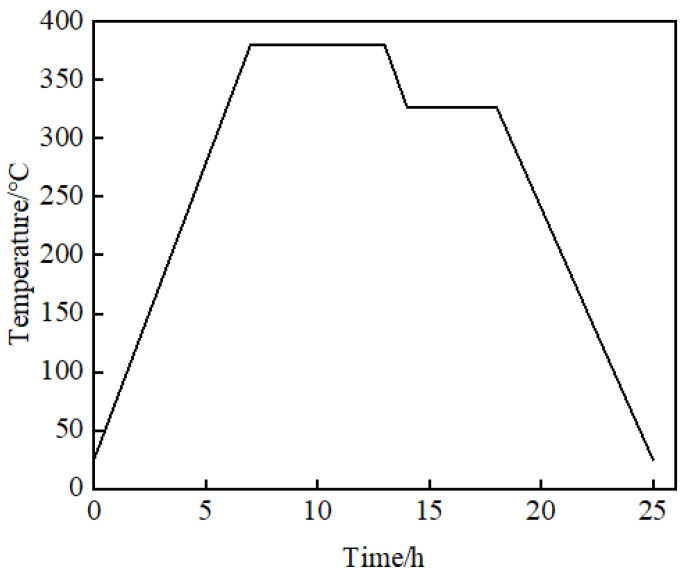
The sintering temperature profile.

**Figure 3:**
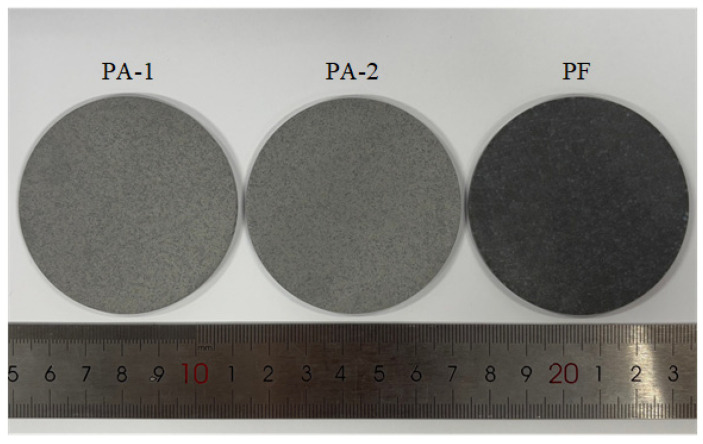
Typical specimens of fluoropolymer-matrix composites after sintering.

The powder mixture was loaded into a steel ball mill with ceramic balls and ball milled for 30 min in a planetary mill. Then powder mixture was placed in a steel mold and cold-pressed at 25 MPa pressure using a hydraulic press, with a holding time of 5 min. The pressed samples were allowed to stand for 24 h to eliminate internal stress, then placed in a programmable tubular vacuum furnace and sintered according to a preset temperature–time profile, which referred to the sintering curve in [4], as shown in Figure 2. Typical specimens after sintering are shown in Figure 3. Each mixture formulation was prepared as two types of specimens: thin discs (Ø60 × (2.5 ± 0.02) mm) and thick discs (Ø60 × 20 mm). In the experiment, a specimen assembly consists of three 2.5 mm specimens for gauges positioning and one 20 mm specimen for delaying the influence of reflected waves generated by the bottom aluminum plate. The 2.5 mm specimens must be prepared with sufficient thickness accuracy to calculate wave velocity precisely.

### 2.2. Plane-Wave Explosive Experiments

**Figure 4:**
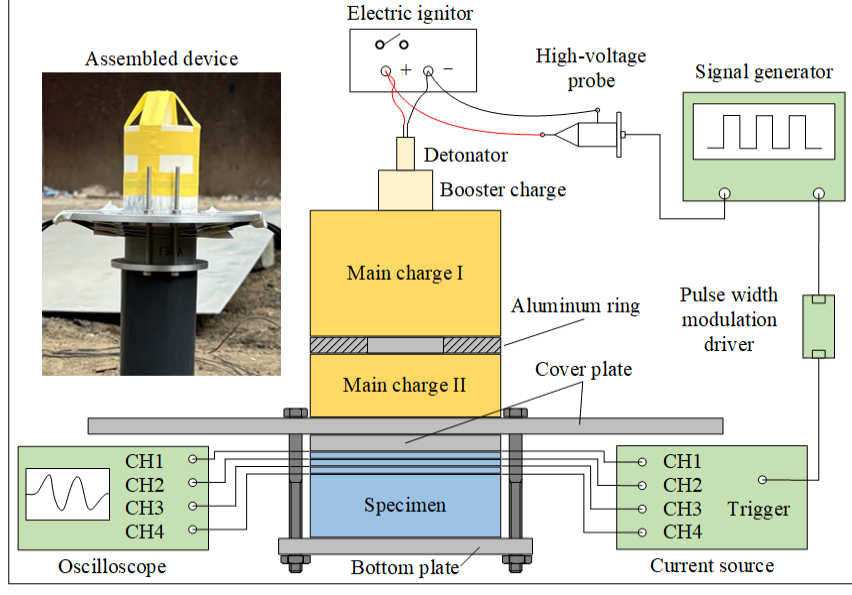
Layout of explosive loading experiment.

Explosive loading experiments were conducted to study the response behavior of materials under high shock pressure. Figure 4 shows the schematic diagram of the experimental setup, which mainly consists of a simple planar wave generator, testing system, and specimens. The simple planar wave generator comprises main charges (main charge I and II) and aluminum rings. The main charge JH-2 explosive was composed of 95 wt% cyclo-1,3,5-trimethylene-2,4,6-trinitramine (RDX), 3 wt% C7H6N2O4 (DNT), and 2 wt% polyvinyl acetate (CZ), with a density of 1.65 ± 0.02 g/cm^3^ pressed with a pressing-explosive machine, and a Chapman–Jouguet pressure of approximately 29.5 GPa. All explosives were from the same batch for experiments and had dimensions of Ø60 × (40 ± 0.5) mm and Ø60 × (20 ± 0.5) mm. The main charge was detonated by an electric detonator and a booster explosive (desensitized hexogen, Ø20 × (20 ± 0.5) mm, density 1.65 ± 0.02 g/cm^3^).

**Figure 5:**
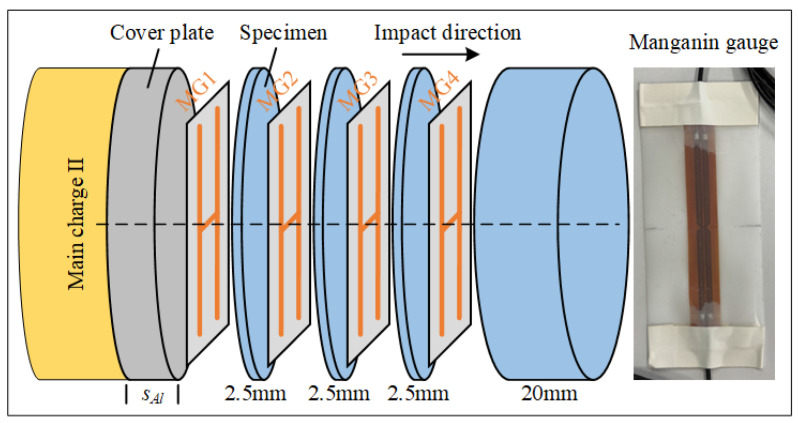
Assembly schematic diagram of specimen embedded with film manganin gauge.

Figure 5 shows the assembly schematic diagram of specimens and film manganin gauges (MGs). Three 2.5 mm-thick specimens and one 20 mm-thick specimen are assembled together, with the thin specimens used to delineate internal positions and the thick specimen used to avoid the influence of reflected waves from the specimen bottom on measurement results. The separator plate, manganin gauges, and specimens are assembled sequentially, with the interface between the upper aluminum separator plate and specimen designated as the loading interface at *x* = 0 mm. To increase the effective testing time of the manganin gauge under extreme conditions, polytetrafluoroethylene films with thickness of 0.1 mm are used to encapsulate the manganin gauges. Prior to assembling the specimens and sensors, the specimen surfaces were cleaned with ethanol to avoid contamination effects. At all interfaces shown in the figure, an appropriate amount of silicone grease is applied and compressed to ensure no air bubbles exist between interfaces. The pressure-sensitive area of the H-type manganin gauge needs to be carefully positioned at the center of the specimen. The thickness of the aluminum separator plate between the explosive and specimen is *s_Al_*, and the incident shock pressure amplitude at the loading interface is adjusted by changing the separator plate thickness.

The output signal from the electric ignitor is transmitted through a high-voltage probe, signal generator, and pulse width modulation driver to synchronously trigger the fast-response pulsed current source (MH2012, Beijing Institute of Technology, Beijing, China). The current source establishes a stable constant current pulse in the gauge within tens of nanoseconds.

The manganin gauge (Beijing Institute of Technology, Beijing, China) has an effective pressure range of 1.5–41.7 GPa. When the shock wave generated by the explosive reaches the position of the manganin gauge, it first causes a significant and rapid pressure jump, followed by a pressure decay process. Over time, the manganin gauge and circuit will be damaged by the shock wave, causing strong interference or interruption of the signal. For manganin gauges embedded at different positions within the specimen, a set of voltage–time variation curves will be recorded by a oscilloscope. The oscilloscope (MDO4104C, Tektronix, Beaverton, OR, USA) features a sampling frequency of 1.5 GHz and achieves a time resolution better than 1 ns. It can be converted to corresponding loading pressure data based on the piezoresistive calibration relationship as follows [24]:

(1) $$P=k_{0}+k_{1}\frac{\Delta R}{R_{0}}+k_{2}(\frac{\Delta R}{R_{0}}{)}^{2}$$

(2) $$\frac{\Delta R}{R_{0}}=\frac{\Delta V}{V_{0}}$$

where *P* is pressure (GPa), *R*_0_ = 0.1–0.2 Ω, and Δ*R* are the electric resistance and its increment, respectively, of the gauge caused by the piezo-resistive effect during the experiments. The coefficients *k*_0_, *k*_1_, and *k*_2_ are 0.76356 ± 0.1811, 34.62796 ± 0.96071, and 6.00762 ± 0.97841, respectively. Moreover, *V*_0_ and Δ*V* are the corresponding voltage and its increment, respectively, across the gauge recorded by the oscilloscope.

Combining Equations (1) and (2) and taking the partial derivative with respect to *P*, the pressure uncertainty is obtained as follows:

(3) $$\delta P=(k_{1}+2k_{2}\frac{\Delta V}{V_{0}})\times \delta (\frac{\Delta V}{V_{0}})$$

where the voltage measurement uncertainty from the oscilloscope is as follows:

(4) $$\delta (\frac{\Delta V}{V_{0}})=\;\frac{\Delta V}{V_{0}}\sqrt{{\left(\frac{\delta (V_{0})}{V_{0}}\right)}^{2}+{\left(\frac{\delta (\Delta V)}{\Delta V}\right)}^{2}}$$

where δ(*V*_0_) and δ(Δ*V*) are the uncertainties of voltage and its increment. Under the measurement range and oscilloscope settings used in this experiment, the oscilloscope has a voltage measurement error of less than 3%.

**Figure 6:**
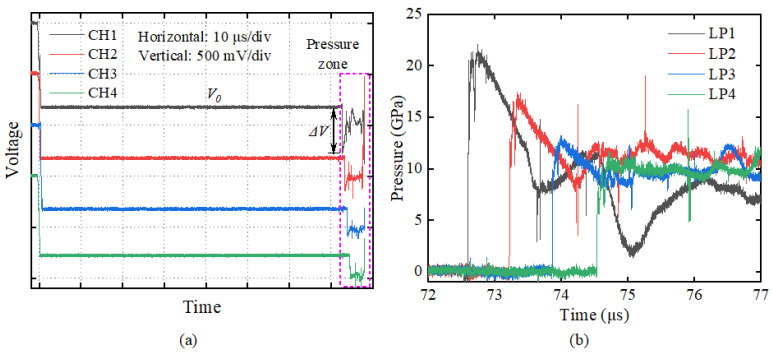
Typical experimental data of Exp. 7#: (**a**) voltage signals recorded on the digital oscilloscope and (**b**) pressure profiles at different positions.

Typical experimental data are shown in Figure 6. The voltage signals recorded by the oscilloscope on channels CH1–CH4 are converted to pressure histories at four Lagrangian locations LP1–LP4 using Equation (1). The manganin gauge has a reference voltage of approximately 800 ± 20 mV during testing, with a voltage increment range of 160–460 mV corresponding to the measurement range of 8.0–22.6 GPa, resulting in a final pressure measurement uncertainty of less than 5%. All experimental data are presented in Section 3.1 for convenient comparison.

## 3. Results and Analysis

### 3.1. Experimental Results and Comparison

**Figure 7:**
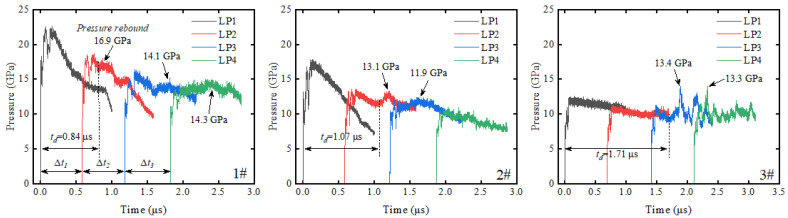
Pressure profiles at four Lagrangian positions in PTFE/Al materials (PA-1).

**Figure 8:**
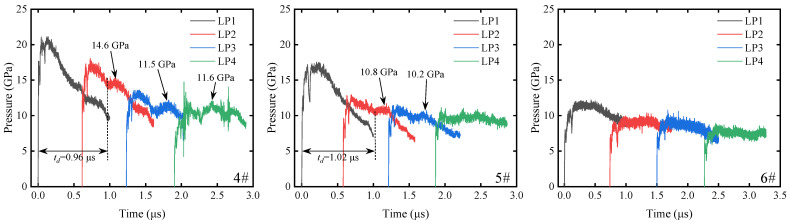
Pressure profiles at four Lagrangian positions in PTFE/Al materials (PA-2).

**Figure 9:**
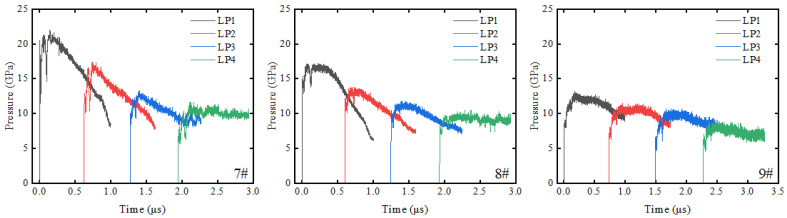
Pressure profiles at four Lagrangian positions in PTFE/LiF materials.

In this study, a total of nine groups of explosive loading experiments were conducted, using 10/16/25 mm aluminum plates to adjust the incident shock pressure at the material loading interface. As illustrated in Figure 6b, the reflected wave generated by the aluminum attenuator reaches the gauge approximately 1 μs following the arrival of the initial shock wave front. Figure 7, Figure 8 and Figure 9 present the pressure profiles at four Lagrangian positions in PA-1, PA-2, and PF, respectively. The duration of the pressure profile at all gauges is limited to 1 μs to ensure exclusion of reflected wave interference. Exp. 1#, 4#, and 7# had incident shock pressures of 21.3–22.6 GPa; Exp. 2#, 5#, and 8# had incident shock pressures of 16.5–17.2 GPa; Exp. 3#, 6#, and 9# had incident shock pressures of 11.5–12.4 GPa.

Compared with the LiF-containing inert material (PF), the aluminum-containing reactive materials (PA-1, PA-2) show obvious pressure rebound phenomena after experiencing initial pressure decay. As shown in Figure 7, PA-1 exhibits pressure rebound under all incident shock pressures tested. The maximum pressure rebound values in Exp. 1#, 2#, and 3# are 16.9 GPa, 13.4 GPa, and 13.1 GPa, respectively. As shown in Figure 8, PA-2 shows maximum pressure rebound values of 14.6 GPa and 10.8 GPa in Exp. 4# and 5#, respectively, while no pressure rebound is observed in Exp. 6# under lower incident shock pressure. As shown in Figure 9, no pressure rebound is observed in PTFE/LiF, showing only a “clean” unloading stage. These results indicate that the aluminum powder and PTFE undergo intense oxidation-reduction reactions under the action of the initial shock wave, and the reaction process releases a large amount of chemical energy, altering the pressure unloading curves of the materials.

In the inert PTFE/LiF experiments, the pressure curves at LP4 exhibit relatively small fluctuations. These minor fluctuations are distinctly different from the pressure rebounds caused by chemical reactions. In some reactive PTFE/Al experiments, such as Exp. 1# and 4#, there is an absence of significant pressure rebound at LP4, but with increased oscillation in pressure curve fluctuations. Furthermore, pressure rebounds are barely distinguishable at LP4 in Exp. 2# and 5#. Therefore, distinct pressure rebounds are considered as evidence of reactions, while minor fluctuations or the absence of pressure rebounds are excluded from reaction evidence.

As the shock wave propagates through the material, both shock front values and pressure rebound values attenuate with increasing depth at four Lagrangian positions within the material. In Exp. 1# and 4#, pressure rebounds at LP2 and LP3 positions are significant, while pressure rebound values at LP4 approach the pressure of the initial shock front. In Exp. 2# and 5#, pressure rebounds at LP2 and LP3 positions become less pronounced due to decreased incident shock pressure. At LP4, pressure curves show negligible pressure rebound compared with the inert material in Exp. 8#. The decreasing trend of pressure rebounds suggests that the energy released by the reaction dissipates during propagation behind the shock front.

**Figure 10:**
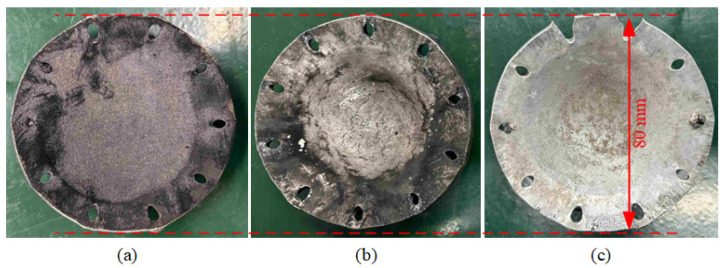
Bottom aluminum plates recovered on site: (**a**) Exp. 3# PA-1, (**b**) Exp. 6# PA-2, and (**c**) Exp. 9# PF.

Bottom aluminum plates from low incident pressure experiments were recovered on site. As shown in Figure 10, aluminum plate surfaces were blackened in Exp. 3# and 6# due to direct contact with PTFE/Al materials and adherent reaction products. Conversely, the aluminum surface remained pristine with no evidence of black residue deposition in Exp. 9#, indicating that the inert PTFE/LiF materials do not react under shock compression. Thus, the blackened plates in this study show similar surface conditions to the recovered plates in reference [16], providing evidence of material reaction.

In this study, a delay time *t_d_* is defined, which describes the time interval from incident shock front arrival to the first observed pressure perturbation induced by reaction. The delay time of PTFE/Al materials shows an increasing trend as the incident shock pressure decreases. The delay times of PA-1 and PA-2 are similar in Exp. 1#, 2#, 4#, and 5#, ranging from 0.84 to 1.07 μs, respectively. When the incident pressure further decreases, the delay time of PA-1 in Exp. 3# increases to 1.71 μs, while no reaction-induced pressure perturbation is detected for PA-2 in Exp. 6#. However, considering the reaction evidence adhering to the recovered bottom aluminum plate surface in Exp. 6#, PA-2 did indeed react, but the pressure perturbation was not detected because the delay time was outside the effective measurement window of the gauge. In Exp. 6#, the measurement windows of gauges at various Lagrangian positions overlap with each other, and none show significant pressure perturbations, indicating that the delay time may exceed 3 μs.

### 3.2. Validation by Hugoniot Data

The experimental parameters and Hugoniot data are summarized in Table 2. *P_i_* represents the peak pressure values measured by the manganin gauge, where subscript *i* takes values 1–4, corresponding to four Lagrangian positions (LP1–LP4) at *x* = 0, 2.5, 5, and 7.5 mm, respectively. Based on the time difference Δ*t_j_* of shock wave arrival at the manganin gauge on the upper and lower surfaces of each specimen layer and the specimen thickness *s_j_*, the average shock velocity in that specimen layer is calculated as *u_sj_ = s_j_*/Δ*t_j_*, where subscript *j* takes values 1–3, corresponding to the first to third specimen layers, respectively. The tolerance range of us is 27–37 m·s^−1^, determined based on specimen thickness and shock front time interval. According to the Hugoniot relationship, the expressions for shock pressure *P*, shock velocity *u_s_*, and particle velocity *u_p_* are as follows [29]:

(5) $$P=\rho_{0}u_{s}u_{p}$$

(6) $$u_{p}=-\rho_{0}u_{s}(v_{1}-v_{0})$$

where *ρ*_0_ is the initial density, and *v*_0_ and *v*_1_ are the initial specific volume and the specific volume after shock compression, respectively. All Hugoniot parameters listed in Table 2 are obtained through calculations using the above relationships.

**Figure 11:**
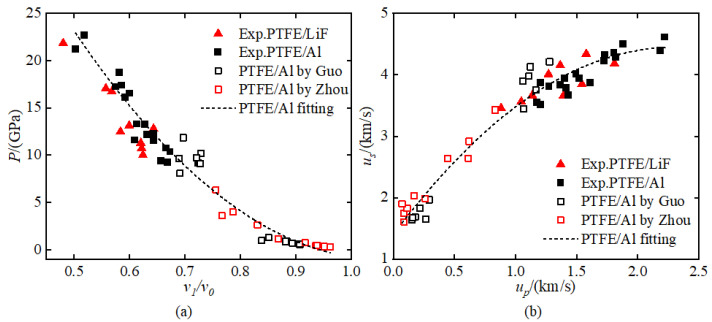
Hugoniot data obtained in this study and reference data from Zhou [11] and Guo [20]: (**a**) *P*–*v*_1_*/v*_0_ and (**b**) *u_s_*–*u_p_*.

Figure 11 shows the Hugoniot relationship scatter plots for different materials, including the pressure-relative specific volume relationship (*P*–*v*_1_*/v*_0_) and the shock wave velocity–particle velocity relationship (*u_s_*–*u_p_*). The experimental results for PTFE/LiF and PTFE/Al in the figure are from the experimental data in this paper, while the reference data are from Zhou [11] (gas gun) and Guo [20] (gas gun and explosive loading experiments). The black dashed lines represent the fitting results for PTFE/Al materials. In the *P*–*v*_1_*/v*_0_ relationship plot (Figure 11a), the compression characteristic for PTFE/LiF and PTFE/Al composites show essentially consistent trends, indicating that the two materials have similar compressibility. In the *u_s_*–*u_p_* relationship plot (Figure 11b), all materials show consistent trends in shock wave velocity–particle velocity relationships. The experimental results indicate that the shock compression responses of PTFE/Al and PTFE/LiF showed no significant differences under the tested conditions.

Although the pressure measurements for each material were obtained from single experiments with relatively limited data sets, comparative analysis against established literature values demonstrates excellent agreement with previously reported results. This consistency validates the experimental pressure data obtained in this investigation. The experimental results of this study supplement the shock compression characteristic data for different pressure ranges and material formulations.

### 3.3. Effect of Particle Size and Pressure on Pressure Impulse

Pressure impulse (*PI*), representing the integral of pressure over time, serves to characterize the intensity properties of shock pressure waves [30,31]. In this study, *PI_x_* is defined the pressure impulse at Lagrange position *x*, with the expression shown as follows:

(7) $${PI}_{x}=\int P_{x}(t)dt$$

where *P_x_*(*t*) represents the pressure–time profile at Lagrangian position *x*. The pressure curves from Section 3.1 are used in the calculations, and the calculation results are listed in Table 3.

For convenient comparative analysis, relative pressure impulse is calculated to eliminate the systematic influence of incident pressure amplitude fluctuations. The relative pressure impulse, *PI_x_*/*PI*_1_, represents a dimensionless ratio of the pressure impulse value *PI_x_* at each Lagrangian position to the incident pressure impulse value *PI*_1_.

**Figure 12:**
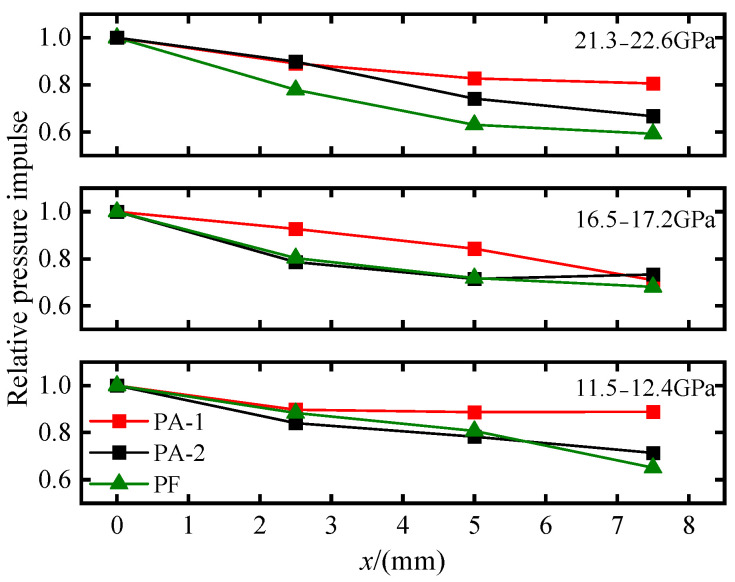
Relative pressure impulse (*PI_x_*/*PI*_1_) at four Lagrangian positions.

As shown in Figure 12, with increasing propagation distance, the relative pressure impulse of different materials all exhibit attenuation, but with different attenuation trends. Under different incident shock pressures, the relative pressure impulse of PA-1 is higher than that of PA-2. Moreover, as shock wave propagation distance increases, PA-1 exhibits slower attenuation velocity than PA-2 at most measurement points, indicating that reducing aluminum particle size effectively enhances rapid energy release in PTFE/Al under shock compression. When incident shock pressure gradually increases, the attenuation velocity of relative pressure impulse for PA-1 at different measurement points shows minimal sensitivity to pressure variations. In contrast, the attenuation velocity for PA-2 gradually slows down with increasing pressure. This indicates that PTFE/Al with small-sized aluminum particles can easily react under relatively low shock pressures, with a lower ignition threshold compared to materials with large-sized aluminum particles.

Since no reaction occurs in PTFE/LiF during shock compression, energy is irreversibly dissipated during shock wave propagation, and the attenuation process of pressure impulse results reflects the shock wave transmitting process. However, when reaction is triggered in PTFE/Al, energy is released, which has a supplementary effect on the energy dissipation of the shock wave, slowing down the attenuation of pressure impulse. Based on the degree of dispersion between the relative pressure impulse of PTFE/Al and PTFE/LiF, it can be inferred whether PTFE/Al has reacted within the measurement time window of that gauge. The closer to the PTFE/LiF results, the closer the reaction is to zero. When it is impossible to determine whether a reaction has occurred through pressure perturbation induced by reaction, pressure impulse serves as a supplementary means. Under low shock pressure, the relative pressure impulse results of PA-2 are close to those of PF, suggesting that PA-2 material did not undergo reaction within the measurement time window.

## 4. Discussion

**Figure 13:**
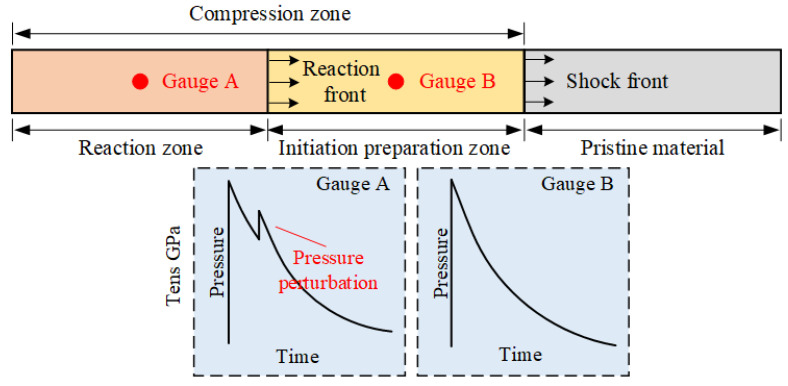
Schematic of reaction behavior during shock compression.

As illustrated in the schematic diagram of Figure 13, the material undergoes an initiation preparation period before the reaction is triggered in the compression zone behind the shock front. The passage of both the shock front and reaction front causes changes in the material state at the gauge location. The time interval of the reaction zone corresponds to the delay time observed in the experiments. The time interval of the shock initiation preparation zone corresponds to the gap between the shock front and the subsequent pressure perturbation induced by reaction. Under relatively high shock pressure, the material undergoes a very short initiation preparation period after shock front passage, and then the reaction is triggered rapidly. Mesoscale numerical simulations reveal that significant temperature gradients develop between PTFE and Al components shortly after shock wave [11]. This temperature difference is attributed to mechanical properties between component materials. PTFE more readily absorbs shock compression energy and consequently achieves higher temperatures. Under high-pressure conditions, aluminum particles may directly reach their melting point, which is commonly regarded as the ignition threshold for aluminum. During intense compression, the oxide layer on the surface of aluminum particles is disrupted, exposing sufficient reactive aluminum. Strong compression also significantly promotes PTFE decomposition. These favorable factors greatly facilitate overcoming reaction activation energy barriers and achieving immediate chemical reaction initiation, which is consistent with the rapid initiation characteristics of reactions under high-pressure conditions observed in this study (Exp. 1#, 2#, 3#, 4#).

Pressure perturbation caused by reactions were almost all observed behind the shock front in this study, except at LP4 in Exp. 3# under low shock pressure, where the pressure perturbation “caught up” with the shock front. In referenced experiments [20], due to the use of smaller aluminum particles (5 μm) in reactive materials, similar pressure perturbation “caught up” with the shock front even under lower incident shock pressure (10.42 GPa). However, in referenced experiments [16], reactive materials with 10 μm aluminum particles under 13.4 GPa showed that pressure perturbations did not catch up with the shock front. This interaction between pressure disturbances and the shock front may be related to reaction rate. When the energy release rate of reaction under shock compression is sufficiently fast, the propagation speed of state changes in the compressed material is fast enough to affect the slower-propagating shock front. However, under the same particle size conditions at higher shock pressures, even though the reaction energy release rate is equally fast, the reaction-induced pressure disturbances can only lag behind the shock front and cannot “catch up” due to the faster propagation speed of the shock front. This may indicate that the energy released by fine aluminum particles can enhance shock front under low shock pressure. Notably, in some porous PTFE/Al composites, researchers have observed pressure growth in shock wave propagation [32]. These indicate that there is partial self-sustaining capability in PTFE/Al under specific conditions.

It was found that the pressure perturbation amplitude induced by reaction gradually attenuated with increasing propagation distance. This characteristic indicates that the reaction of aluminum-containing reactive materials is not self-sustaining, with limited reaction zones that cannot continuously provide energy supplementation to the shock wave front. Only pressure perturbation induced by the reactions of materials activated under initial shock loading can be detected by downstream measurement points. As the propagation distance increases, the reaction intensity gradually weakens, and pressure perturbation is attenuated. The energy released by chemical reactions of reactive materials undergoes dissipation, hardly enhancing the shock front, leading to a continuous decline in shock pressure peaks. This reaction characteristic is fundamentally different from the detonation of explosives. In explosives, the reaction zone closely follows the initial shock wave front, and continuous chemical reactions can maintain the intensity of the shock wave, whereas in aluminum-containing reactive materials, the reaction manifests more as a localized, discontinuous chemical energy release process.

Thadhani [33] proposed two mechanisms: shock-induced chemical reaction (SICR) and shock-assisted chemical reaction (SACR). SICR occurs at or near the shock front, where materials are in a highly compressed state. The reaction duration ranges from nanoseconds to tens of microseconds, but the released energy and material expansion are insufficient to drive self-sustaining shock wave propagation in RMs. SACR occurs at unloading after shock compression, where shock waves primarily provide favorable reaction conditions such as increasing material temperature, activating reactants, and promoting material mixing. The reaction duration is approximately tens of milliseconds. Under shock compression at incident pressures of tens of GPa, the PTFE/Al material’s reaction behavior approaches SICR. When loading intensity further decreases to MPa levels, as in SHPB tests [6] and drop weight tests [19], flame flash typically appears in fragment cloud regions after specimen fragmentation, with delay times ranging from tens of microseconds to milliseconds. At this stage, materials have already been subjected to multiple shock wave reflections, loading–unloading cycles, and inter-fragment collision processes. The reaction behavior more closely approaches SACR under these conditions.

The pressure perturbation characteristics induced by reactions in PTFE/Al materials during shock compression vary under different influencing factors, including shock pressure amplitude and aluminum powder particle size. The essence of this phenomenon may stem from the contributions of multiple factors to aluminum ignition. The aluminum powder particle size primarily affects its ignition delay and reaction rate. Generally, smaller aluminum particles exhibit lower ignition thresholds and faster reaction rates, attributed to their larger specific surface area and reaction control mechanisms [34,35]. Under high shock pressure, sufficient shock compression can provide sufficient temperature conditions, effectively destroy material microstructure, and promote adequate contact and mixing between reactants [36]. These can thereby significantly shorten reaction delay time. Conversely, reduced shock pressure leads to insufficient energy deposition within composites, where the shock-induced temperature rise may fail to rapidly trigger ignition, and microscopic damage is weakened, causing obstruction of the reaction initiation process and a significant increase in delay time. The reaction mechanism becomes more complex and may require consideration of synergistic effects from multiple factors, such as frictional heating and heat transfer between components [6,7].

It should be noted that this study was limited by the effective measurement duration of manganin sensors and could not capture reaction behavior data over extended time periods. Future research could improve measurement methods and employ numerical simulations to investigate reaction characteristic throughout complete or multiple shock compression cycles.

## 5. Conclusions

In this study, shock pressure–time profiles inside Al-containing reactive and LiF-containing inert materials were obtained through explosive shock experiments. The pressure perturbations induced by reaction in reactive materials under shock compression were identified. The effects of different shock pressures and aluminum powder particle sizes on reaction behaviors were investigated. The main conclusions are as follows:

(1) Under high shock pressure, PTFE/LiF remains inert and exhibits similar compression characteristic to PTFE/Al. The reaction-induced pressure perturbations in reactive materials were identified by comparing pressure profiles with those of inert counterparts.
(2) The pressure rebounded to a range of 10.2–16.9 GPa under an incident shock pressure range of 11.5–22.6 GPa. The pressure perturbation amplitude induced by reaction gradually attenuated with increasing propagation distance.
(3) The delay time between the observed pressure perturbations and the incident shock front arrival ranged from 0.84 to 1.71 μs, and showed a decreasing trend with increasing incident shock pressure and decreasing aluminum particle size.
(4) The reaction ignition and energy release of PTFE/Al materials change from closely following the shock front to being delayed by hundreds of microseconds behind the shock front when shock compression intensity decreases from GPa to MPa levels.

## Figures and Tables

**Table 1:** Details of specimen preparation.

No.	Formula	Mass Ratio/(wt%)	Theoretical Density /(g·cm^−3^)	Actual Density /(g·cm^−3^)	Relative Density	Particle Size /(μm)
PA-1	PTFE/Al	73.5/26.5	2.27	2.23	98%	25/25
PA-2	PTFE/Al	73.5/26.5	2.27	2.23	98%	25/75
PF	PTFE/LiF	73.5/26.5	2.26	2.21	98%	25/25

**Table 2:** (**a**). The experiments parameters and related results. (**b**). The experiment parameters and related results.

(**a**). The experiment parameters and related results.															
**Exp. No.**		**Specimen**		***s_Al_*** **/(mm)**		***P_i_*/(GPa)**									
						***P*_1_**			***P*_2_**		***P*_3_**			***P*_4_**	
1#		PA-1		10		22.6 ± 1.05			18.7 ± 0.86		16.1 ± 0.74			13.7 ± 0.64	
2#				16		17.2 ± 0.79			13.2 ± 0.62		11.5 ± 0.55			10.2 ± 0.49	
3#				25		12.1 ± 0.57			10.3 ± 0.51		9.1 ± 0.45			12.6 ± 0.59	
4#		PA-2		10		21.3 ± 0.99			17.3 ± 0.80		13.2 ± 0.62			11.2 ± 0.53	
5#				16		16.5 ± 0.76			12.2 ± 0.57		10.7 ± 0.51			9.9 ± 0.48	
6#				25		11.5 ± 0.54			9.2 ± 0.45		9.3 ± 0.46			9.1 ± 0.44	
7#		PF		10		21.8 ± 1.01			17.0 ± 0.78		12.8 ± 0.60			10.7 ± 0.52	
8#				16		16.7 ± 0.77			13.1 ± 0.61		11.2 ± 0.53			9.2 ± 0.45	
9#				25		12.4 ± 0.58			10.6 ± 0.51		10.0 ± 0.49			8.0 ± 0.41	
(**b**). The experiment parameters and related results.															
**Exp. No.**	***u_sj_*/(m** **·s^−1^)**						***u_pj_*/(m** **·s^−1^)**					**Relative Volume *v*_1_*/v*_0_**			
	***u_s_*_1_**		***u_s_*_2_**		***u_s_*_3_**		***u_p_*_1_**	***u_p_*_2_**		***u_p_*_3_**		***LP1***	***LP2***		***LP3***
1#	4611		4500		4230		2222	1880		1724		0.5181	0.5821		0.5925
2#	4283		3945		3832		1819	1524		1364		0.5753	0.6136		0.6439
3#	3870		3785		3869		1424	1237		1070		0.6321	0.6731		0.7234
4#	4389		4353		4014		2185	1805		1495		0.5023	0.5853		0.6275
5#	4325		3942		3812		1729	1403		1273		0.6002	0.6440		0.6660
6#	3662		3546		3509		1430	1177		1205		0.6094	0.6681		0.6564
7#	4340		4156		4010		2256	1840		1431		0.4801	0.5571		0.6429
8#	4185		3850		3661		1808	1542		1393		0.5678	0.5993		0.6194
9#	3665		3563		3456		1527	1344		1299		0.5834	0.6226		0.6240

**Table 3:** Pressure impulse calculation results.

Exp. No.	*PI_x_*/(10^3^ Pa·s)			
	*PI*_1_	*PI*_2_	*PI*_3_	*PI*_4_
1#	15.95	14.20	13.19	12.86
2#	12.44	11.52	10.49	8.83
3#	11.01	9.88	9.77	9.78
4#	14.62	13.12	10.84	9.75
5#	12.52	9.84	8.94	9.17
6#	10.19	8.57	7.98	7.27
7#	15.95	12.42	10.06	9.45
8#	13.15	10.56	9.44	8.94
9#	10.93	9.66	8.83	7.12

## Data Availability

The original contributions presented in this study are included in the article. Further inquiries can be directed at the corresponding authors.
